# Luminescence and luminescence quenching of highly efficient Y_2_Mo_4_O_15_:Eu^3+^ phosphors and ceramics

**DOI:** 10.1038/srep26098

**Published:** 2016-05-16

**Authors:** Matas Janulevicius, Paulius Marmokas, Martynas Misevicius, Julija Grigorjevaite, Lina Mikoliunaite, Simas Sakirzanovas, Arturas Katelnikovas

**Affiliations:** 1 Department of Analytical and Environmental Chemistry, Vilnius University, Naugarduko 24, LT-03225 Vilnius, Lithuania; 2 Department of Inorganic Chemistry, Vilnius University, Naugarduko 24, LT-03225 Vilnius, Lithuania; 3 Department of Physical Chemistry, Vilnius University, Naugarduko 24, LT-03225 Vilnius, Lithuania; 4 Department of Applied Chemistry, Vilnius University, Naugarduko 24, LT-03225 Vilnius, Lithuania; 5 Institute of Chemistry, Centre for Physical Sciences and Technology, A. Gostauto 9, LT-01108 Vilnius, Lithuania

## Abstract

A good LED phosphor must possess strong enough absorption, high quantum yields, colour purity, and quenching temperatures. Our synthesized Y_2_Mo_4_O_15_:Eu^3+^ phosphors possess all of these properties. Excitation of these materials with near-UV or blue radiation yields bright red emission and the colour coordinates are relatively stable upon temperature increase. Furthermore, samples doped with 50% Eu^3+^ showed quantum yields up to 85%, what is suitable for commercial application. Temperature dependent emission spectra revealed that heavily Eu^3+^ doped phosphors possess stable emission up to 400 K and lose half of the efficiency only at 515 K. In addition, ceramic disks of Y_2_Mo_4_O_15_:75%Eu^3+^ phosphor with thickness of 0.71 and 0.98 mm were prepared and it turned out that they efficiently convert radiation of 375 and 400 nm LEDs to the red light, whereas combination with 455 nm LED yields purple colour.

Solid state light sources based on the blue emitting InGaN semiconductor chips became a revolution in lighting industry after the discovery of efficient blue emitting diode by S. Nakamura in 1991^1^. However, the blend of the blue light emitted by diode and yellow emitted by a Y_3_Al_5_O_12_:Ce^3+^ (YAG:Ce) phosphor usually yields a cold white light due to deficiency of the red component in the spectrum. To overcome this issue some red phosphors are added to the light source. Frequently used nitride based red phosphors are very expensive and require complicated synthesis techniques. Another way to produce solid state white light sources is to employ near UV emitting LED chip and coat it with red, green and blue phosphor. The advantage of such approach is much broader phosphor selection than for blue LED excitation. Inorganic materials doped with rare earth ions are mostly used as activators in the mentioned phosphors. Since there are some efficient blue (BaMgAl_10_O_17_:Eu^2+^) and green (SrSi_2_O_2_N_2_:Eu^2+^, Ba_2_SiO_4_:Eu^2+^)^2^,^3^ phosphors, the main problems arise with finding a suitable and relatively inexpensive red-emitting phosphor. Moreover, the requirements for LED phosphors are also high, for instance, strong absorption of LED radiation, high thermal quenching temperature, high quantum yield, excellent chemical and thermal stability and absence of emission saturation at high fluxes^4^. Unfortunately, it is very hard to find materials that meet all the aforementioned criteria and, therefore, the reports of efficient Eu^3+^ doped phosphors with high thermal stability is scarce.

Trivalent europium doped materials are usually considered as good red-emitting phosphor candidates for LEDs. On the other hand, Eu^3+^ ions typically possess rather low absorption strength due to the spin and parity forbidden nature of their intraconfigurational [Xe]4f^6^ → [Xe]4f^6^ transitions^5^. However, in molybdates, tungstates, niobates and vanadates these transitions, especially at shorter wavelengths (<400 nm), become rather strong due to admixing with low lying charge transfer (CT) band^6^,^7^,^8^. The position of the CT band depends on the host material as do the emission spectra of Eu^3+^ ions. Thus by selecting the appropriate host material one should be able to obtain a desired absorption strength and emission profile.

Furthermore, the rare earth doped molybdates and tungstates also attract much attention for application in areas such as lasers^9^, scintillators^10^, upconverters^11^, and bio-imaging^12^ to mention just a few. This shows the versatility of molybdate and tungstate based compounds. Thus the research of these compounds is very intensive and new structures are reported every so often^11^,^13^.

In this work, Y_2_Mo_4_O_15_:Eu^3+^ compounds were investigated as a potential phosphors for blue and near-UV LEDs. These materials showed good colour saturation, high luminous efficacies, and high external quantum yields. Phosphors also possessed high thermal quenching temperature what is favourable in application with high power LEDs.

## Experimental

Y_2_Mo_4_O_15_:Eu^3+^ (where Eu^3+^ concentration is 0%, 1%, 5%, 10%, 25%, 50%, and 75%) powders were synthesized by high temperature solid state reaction. The stoichiometric amounts of Y_2_O_3_ (99.99% Tailorlux), Eu_2_O_3_ (99.99% Tailorlux), and MoO_3_ (99+% Acros Organics) were thoroughly mixed in an agate mortar employing some acetone as the grinding medium. The blends of starting materials were dried, transferred to the porcelain crucibles and sintered at 700 °C for 12 h in air. An attempt to synthesize isostructural Eu_2_Mo_4_O_15_ under the same conditions failed.

The Y_2_Mo_4_O_15_:75%Eu^3+^ ceramic disks with a thickness of 0.71 and 0.98 mm were prepared by applying 30 kN force (ø 8 mm disk) on the phosphor powder for 3 min. Subsequently, the obtained pellets were placed on alumina plate and sintered at 700 °C for 4 h in air.

Powder XRD data for phase identification were collected in the range 10° ≤ 2θ ≤ 80° (step width 0.02° and scanning speed 5°/min.) using Ni-filtered Cu Kα radiation on a Rigaku MiniFlexII diffractometer.

Powder XRD data for Rietveld refinement were collected in the range 10° ≤ 2θ ≤ 100° (step width 0.01° and integration time 3 s) using Ni-filtered Cu Kα radiation on a Bruker D8 Advance diffractometer with Bragg-Brentano focusing geometry and position sensitive LynxEYE detector. The Rietveld refinement was carried out on FullProf Suite Program (3.00) software.

FTIR spectra were taken with PerkinElmer Frontier ATR-FTIR Spectrometer equipped with a liquid nitrogen cooled MCT detector. Step width was 2 cm^−1^ and each measurement consisted of 25 scans.

The SEM pictures of phosphor powders and ceramic disks were taken with FE-SEM Hitachi SU-70. The accelerating voltage was 2 kV.

Reflection spectra were recorded on an Edinburgh Instruments FLS980 spectrometer equipped with double excitation and emission monochromators, 450 W Xe arc lamp, a cooled (–20 °C) single-photon counting photomultiplier (Hamamatsu R928) and Teflon coated integration sphere. Teflon was used as a reflectance standard. The excitation and emission slits were set to 3.00 and 0.15 nm respectively. Step width was 0.5 nm and integration time was 0.4 s.

Excitation and emission spectra were recorded on the Edinburgh Instruments FLS980 spectrometer equipped with double excitation and emission monochromators, 450 W Xe arc lamp, a cooled (–20 °C) single-photon counting photomultiplier (Hamamatsu R928) and mirror optics for powder samples. The photoluminescence emission spectra were corrected by a correction file obtained from a tungsten incandescent lamp certified by NPL (National Physics Laboratory, UK). When measuring emission spectra (λ_ex_ = 290, 393.5 and 465 nm) excitation and emission slits were set to 0.3 and 0.25 nm respectively. When measuring excitation spectra (λ_em_ = 616 nm) excitation and emission slits were set to 0.2 and 0.3 nm, respectively. The excitation spectra were corrected by a reference detector. In both cases step width was 0.5 nm and integration time was 0.4 s.

For thermal quenching (TQ) measurements a cryostat “MicrostatN” from the Oxford Instruments had been applied to the present spectrometer. Liquid nitrogen was used as a cooling agent. The measurements were performed at 77 K and at 100–500 K in 50 K intervals. Temperature stabilization time was 90 s and temperature tolerance was set to ±5 K. During the measurements dried nitrogen was flushed over the cryostat window to avoid the condensation of water at low temperatures on the surface of the window.

The photoluminescence decay kinetics studies were performed on the same FLS980 spectrometer. Xe μ-flash lamp was used as an excitation source. Three excitation wavelengths were 290, 393.5, and 465 nm while emission was monitored at 613 nm.

Quantum yields (QY) were calculated by measuring emission spectrum of the Teflon sample in Teflon coated integration sphere. Three different excitation wavelengths, namely, 290, 393.5 and 465 nm, were used and the respective emission spectra were recorded in ranges 270–800 nm, 370–800 nm, and 440–800 nm. The same measurements were repeated for the phosphor samples. The QY values were obtained employing the equation 1^14^:


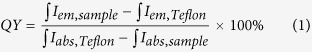


where 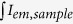 and 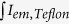 are integrated emission intensities of the phosphor sample and Teflon, respectively. Likewise, 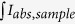 and 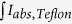 are the integrated absorption of the phosphor sample and Teflon, respectively.

## Results and Discussion

The formula of Y_2_Mo_4_O_15_ compound does not represent it’s actual structure and could be better expressed as Y_2_[MoO_4_]_2_[Mo_2_O_7_]. It is isostructural with Ho_2_[MoO_4_]_2_[Mo_2_O_7_], which was reported by V. A. Efremov *et al*. in 1988^15^. Y_2_Mo_4_O_15_ crystallizes in primitive monoclinic Bravais lattice with the space group P2_1_/c (#14), *Z* = 2. The unit cell is built of isolated [MoO_4_]^2−^ and [Mo_2_O_7_]^2−^ polyhedrons with Y^3+^ ions filling the voids. There is only one crystallographic site for Y^3+^ ions, which are coordinated by seven oxygen ions. The unit cell of Y_2_Mo_4_O_15_ along the axis *b* is shown in Fig. s1.

**Figure 1:**
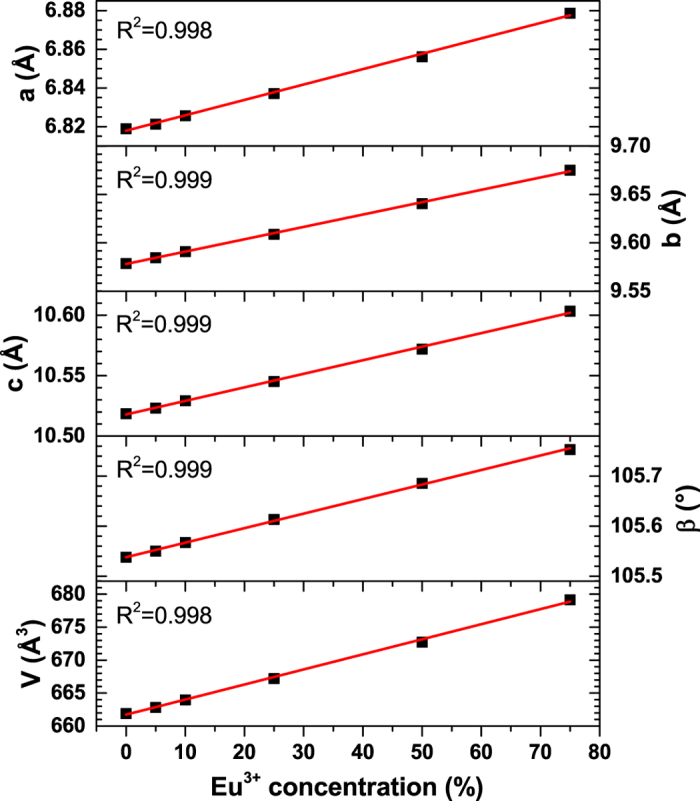
Rietveld refinement data. Y_2_Mo_4_O_15_:Eu^3+^ unit cell parameters as a function of Eu^3+^ concentration.

Synthesis of the Y_2_Mo_4_O_15_:Eu^3+^ powders at 850 °C as reported by S. Laufer *et al*.^16^ yielded a mixture of various yttrium molybdates. On the other hand, single crystals of the title compound in an evacuated silica tube were grown at this temperature. Thus, these synthesis conditions might be inappropriate for the synthesis of powder samples. Lowering synthesis temperature to 700 °C^17^,^18^,^19^ resulted in a single phase Y_2_Mo_4_O_15_:Eu^3+^ powders with Eu^3+^ concentration up to 75%. The XRD patterns of Y_2_Mo_4_O_15_:Eu^3+^ as a function of Eu^3+^ concentration are depicted in Fig. s2, together with the reference pattern for comparison. The finding that solubility of Eu^3+^ ions in Y_2_Mo_4_O_15_ structure is limited to 75% was quite unexpected, since the radii difference of seven coordinated Y^3+^ (0.96 Å) and Eu^3+^ (1.01 Å)^20^ is only around 5%. However, Vegard’s law predicts that a complete solid solution should form if the size difference of ions is in the range of ±15%^21^. This contradiction leads to the conclusion, that Y_2_Mo_4_O_15_ structure is sensitive to the cation size on the Y^3+^ position. Rietveld refinement was performed in order to evaluate the change of lattice parameters upon Eu^3+^ substitution for Y^3+^. The typical refinement graph is shown in Fig. s3, which indicate good agreement between calculated and measured powder XRD patterns. The obtained data are summarized in Fig. 1. As expected the gradual replacement of Y^3+^ by larger Eu^3+^ ions resulted in an increase of all lattice parameters. The increase is linear what is in line with R^2^ values of linear fit of experimental data being close to unity. On the other hand, the absolute change of lattice parameters if 75% Y^3+^ is replaced by Eu^3+^ is quite small: *Δa*, *Δb*, *Δc*, and *Δβ* being 0.9%, 1.0%, 0.8%, and 0.2%, respectively. The exact calculated lattice parameters are listed in Table s1.

The recorded FTIR spectra (see Fig. s4) for undoped sample and samples doped with 10%, 25%, 50%, and 75% Eu^3+^ showed no significant differences between each other. Spectra consisted of several overlapping strong absorption bands in the range of 500–1000 cm^−1^. These bands can be attributed to the characteristic vibrations of [MoO_4_]^2−^ and [Mo_2_O_7_]^2−^ groups^22^,^23^,^24^.

**Figure 2:**
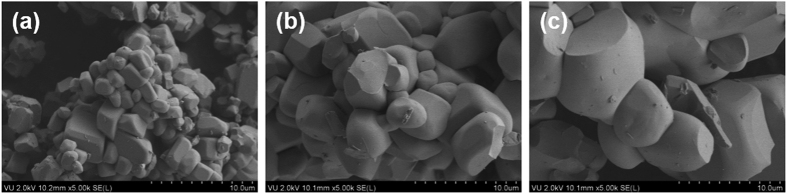
SEM images. (**a**) Y_2_Mo_4_O_15_, (**b**) Y_2_Mo_4_O_15_:50%Eu^3+^, and (**c**) Y_2_Mo_4_O_15_:75%Eu^3+^ under magnification of 5.0 k.

The morphological features of undoped, 50% and 75% Eu^3+^ doped Y_2_Mo_4_O_15_ powder samples were investigated by taking high resolution SEM pictures, which are shown in Fig. 2. The obtained images clearly demonstrate that the particle size of the powders increases with increasing Eu^3+^ content in the structure. Particles are well shaped and their size distribution is rather broad. Moreover, another interesting feature is that there are very little dust particles on top of larger particles. This indicate high powder quality, since no washing or sieving of the obtained powders were performed.

**Figure 3:**
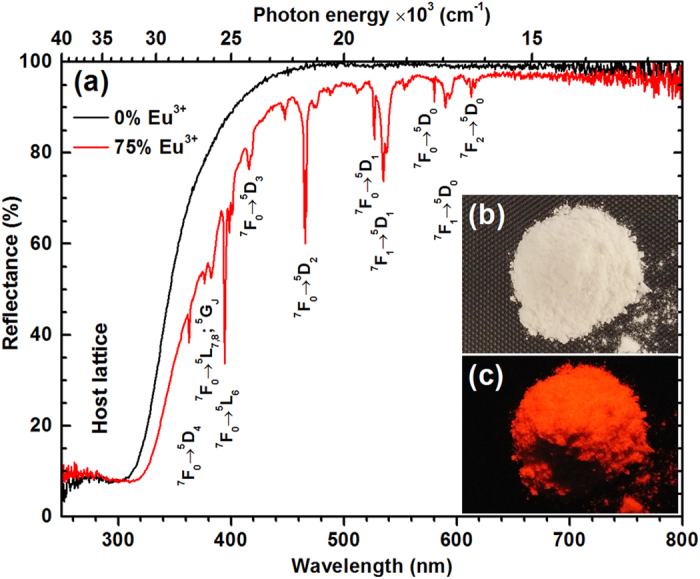
Reflection spectra and digital photos. Reflection spectra of Y_2_Mo_4_O_15_ and Y_2_Mo_4_O_15_:75%Eu^3+^ (**a**), digital photos of Y_2_Mo_4_O_15_ at daylight (**b**) and Y_2_Mo_4_O_15_:75%Eu^3+^ under 254 nm excitation.

The body colour of undoped Y_2_Mo_4_O_15_ powder is white as shown in digital photo in Fig. 3b. White body colour indicates the absence of absorption in the visible range what is in good agreement with reflectance spectrum shown in Fig. 3a. The white body colour of undoped material gradually gained pale rose tint upon increasing Eu^3+^ concentration in the Y_2_Mo_4_O_15_ due to absorption in the violet-blue spectral range causing the luminescence of Eu^3+^ ions^25^. This is all in line with the reflection spectrum of Y_2_Mo_4_O_15_:75%Eu^3+^ shown in Fig. 3a, where typical Eu^3+^ absorption lines originating from the intraconfigurational ^7^F_0_ → ^5^D_J_, ^7^F_0_ → ^5^L_J_ and ^7^F_0_ → ^5^G_J_ transitions^26^,^27^,^28^ are visible. Besides, it is evident that ^7^F_1_ and ^7^F_2_ levels are also thermally populated to some extent since absorption lines originating from the ^7^F_2_ → ^5^D_0_ (ca. 613 nm), ^7^F_1_ → ^5^D_0_ (ca. 590 nm), and ^7^F_1_ → ^5^D_1_ (ca. 527 nm) transitions are clearly visible in the reflection spectra^29^. The broad absorption band at short wavelengths (250–340 nm) can be attributed to the host lattice absorption.

The optical band gap of the undoped, 10%, 25%, 50% and 75% Eu^3+^ materials was evaluated from absorption spectra. Absorption spectra *F*(*R*) were calculated from reflection spectra employing Kubelka-Munk function^17^,^30^:


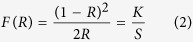


where *R* is reflectance, *K* is absorption coefficient, and *S* is scattering coefficient. It was found out that the energy of optical band gap decreases if the concentration of Eu^3+^ is increased. The band gap estimation is depicted in Fig. s5. The optical band gap energies for undoped, 10%, 25%, 50%, and 75% Eu^3+^ doped Y_2_Mo_4_O_15_ materials are 334 nm (3.71 eV), 336 nm (3.69 eV), 338 nm (3.67 eV), 340 nm (3.65 eV), and 344 nm (3.60 eV), respectively.

**Figure 4:**
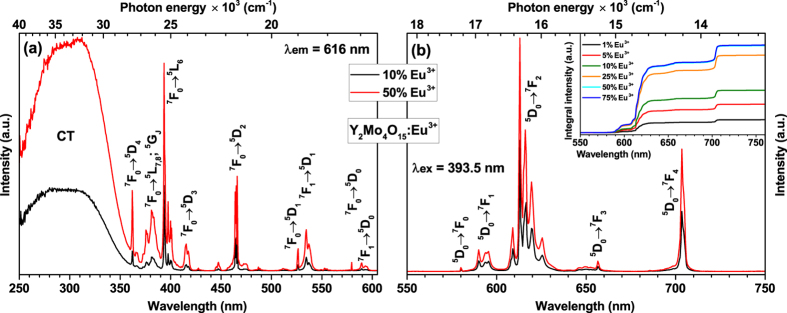
Excitation and emission spectra. (**a**) Excitation (λ_em_ = 616 nm) and (**b**) emission (λ_ex_ = 393.5 nm) spectra of Y_2_Mo_4_O_15_:Eu^3+^ phosphors doped with 10% and 50% Eu^3+^. Inset shows emission (λ_ex_ = 393.5 nm) integral intensity as a function of Eu^3+^ concentration.

Figure 4a shows excitation spectra of 10% and 50% Eu^3+^ doped samples for 616 nm emission. The spectra consist of a broad band in the range of 250–360 nm and several sets of lines in the range of 360–600 nm. The broad excitation band can be attributed to the charge transfer (CT) transition from [MoO_4_]^2−^ and [Mo_2_O_7_]^2−^ groups to Eu^3+^ ions. It was also observed that the energy of charge transfer band slightly decreases (shifts towards longer wavelengths) with increasing Eu^3+^ concentration. This can be explained by the expansion of the lattice leading to the increased average distance between Eu^3+^ and surrounding anions^31^, thus easing the electron transfer from oxygen to europium. The sharp lines are associated with the intraconfigurational [Xe]4f^6^ → [Xe]4f^6^ transitions of Eu^3+^ ions. Excitation lines originating from ^7^F_0_ → ^5^L_6_ (≈394 nm) and ^7^F_0_ → ^5^D_2_ (≈465 nm) are very attractive for application as colour converters in LEDs since they overlap very well with the emission spectra of efficient near-UV and blue LEDs, respectively. All samples doped with Eu^3+^ ions showed bright red luminescence when excited with the UV lamp as indicated in Fig. 3c. The emission spectra of 10% and 50% Eu^3+^ doped samples under 393.5 nm excitation are given in Fig. 4b. The spectra consist of five sets of lines at around 580, 590, 615, 655, and 705 nm, which originate from ^5^D_0_ → ^7^F_0_, ^5^D_0_ → ^7^F_1_, ^5^D_0_ → ^7^F_2_, ^5^D_0_ → ^7^F_3_, and ^5^D_0_ → ^7^F_4_ optical transitions of Eu^3+^ ions, respectively. The intensity of ^5^D_0_ → ^7^F_0_ transition is the weakest since *J* = 0 ↔ *J’* = 0 transitions are always forbidden^32^,^33^,^34^. The most intensive is an electric dipole (ED) ^5^D_0_ → ^7^F_2_ transition, whereas the intensity of a magnetic dipole (MD) ^5^D_0_ → ^7^F_1_ transition is at least by one order of magnitude lower. This indicates that Eu^3+^ ions occupy non-centrosymmetric sites in Y_2_Mo_4_O_15_ host matrix what is in good agreement with the crystallographic data. This is also confirmed by large value (ca. 7.8) of the asymmetry ratio (^5^D_0_ → ^7^F_2_)/(^5^D_0_ → ^7^F_1_), which gives a measure of distortion degree from the inversion symmetry of the local environment of Eu^3+^ ions in the lattice^35^,^36^. Another interesting feature of the emission spectra is oddly intensive emission peak at 705 nm. Such intensive ^5^D_0_ → ^7^F_4_ transitions lines of Eu^3+^ ions are usually observed in some phosphates and garnet structure materials^37^, however, in molybdates they are rather weak^12^,^38^. Nevertheless, the reason of such intensive ^5^D_0_ → ^7^F_4_ transition lines is still unclear and requires additional research.

It is also well known that the emission spectra of Eu^3+^ doped materials might contain the transitions that are overlapping. These transitions are ^5^D_0_ → ^7^F_0_ and ^5^D_2_ → ^7^F_5_, ^5^D_0_ → ^7^F_2_ and ^5^D_1_ → ^7^F_4_, ^5^D_0_ → ^7^F_3_ and ^5^D_1_ → ^7^F_5_^29^. In order to check if any overlapping between the aforementioned transitions is occurring a sample doped with 50% Eu^3+^ was excited at four different wavelengths, namely, 393.5 nm (^7^F_0_ → ^5^L_6_), 465 nm (^7^F_0_ → ^5^D_2_), 534.5 nm (^7^F_1_ → ^5^D_1_), and 589.5 nm (^7^F_1_ → ^5^D_0_). With every increased excitation wavelength (decreased energy) lower and lower excited states of Eu^3+^ were populated. Therefore, if there were any emission from transitions starting at higher energy excited levels that are overlapping with the ones starting at ^5^D_0_ this would be visible in the emission spectra. However, no differences in emission spectra upon changing the excitation wavelength were observed as shown in Fig. s6a. Thus it can be concluded that the emission lines in the range of 580–720 nm originate from the lowest energy excited level ^5^D_0_. On the other hand, some weak emission lines originating from ^5^D_1_, ^5^D_2_ and ^5^D_3_ levels were observed when the 400–580 nm range was highly magnified. These lines are depicted in Fig. s6b,c. The following sets of lines were observed: ^5^D_3_ → ^7^F_1_ (ca. 416 nm), ^5^D_3_ → ^7^F_2_ (ca. 427 nm), ^5^D_3_ → ^7^F_3_ (ca. 446 nm), ^5^D_3_ → ^7^F_4_ and ^5^D_2_ → ^7^F_0_ (ca. 469 nm), ^5^D_2_ → ^7^F_2_ (ca. 486 nm), ^5^D_2_ → ^7^F_3_ (ca. 512 nm), ^5^D_1_ → ^7^F_0_ (ca. 526 nm), ^5^D_1_ → ^7^F_1_ (ca. 535 nm), ^5^D_1_ → ^7^F_2_ (ca. 555 nm). It also needs to be noted that the intensity of the first seven mentioned transitions is by four orders of magnitude lower if compared to the ^5^D_0_ → ^7^F_2_ transition, whereas the intensity of the latter two are by three orders of magnitude lower. This finding confirms that the radiationless decay from the higher energy levels to the ^5^D_0_ level is very effective^29^. The inset in Fig. 4b shows integrated emission spectra and conveys that the strongest emission is observed for the samples doped with 50% and 75% Eu^3+^ (overlapped).

**Figure 5:**
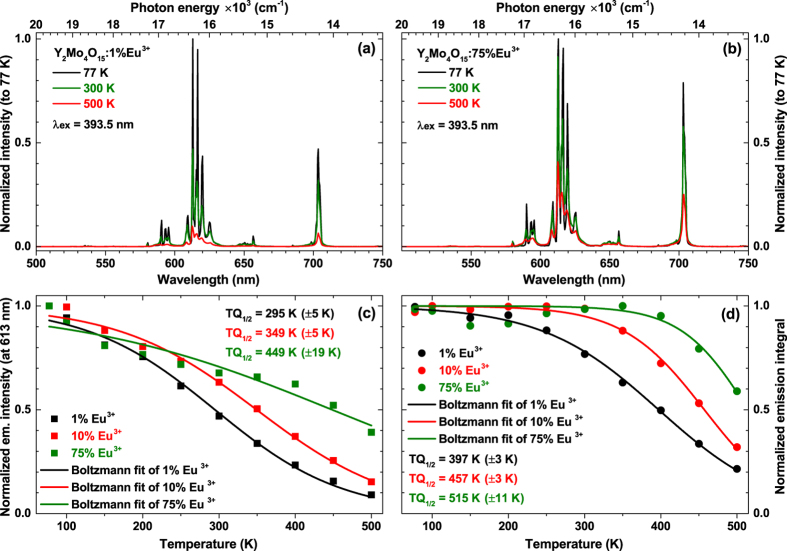
Temperature dependent emission spectra an TQ_1/2_ value evaluation. Temperature dependent emission (λ_ex_ = 393.5 nm) spectra of (**a**) Y_2_Mo_4_O_15_:1%Eu^3+^ and (**b**) Y_2_Mo_4_O_15_:75%Eu^3+^. Calculation of TQ_1/2_ values for the samples doped with 1%, 10% and 75% Eu^3+^ from normalized emission intensity (at 613 nm) (**c**) and normalized emission integral (**d**).

The emission spectra (λ_ex_ = 393.5 nm) of samples doped with 1% and 75% Eu^3+^ as a function of temperature are depicted in Fig. 5a,b, respectively. It is evident that the emission intensity of sample doped with 1% Eu^3+^ decreases much faster upon temperature increase if compared to the 75% Eu^3+^ doped sample. It is also interesting to note that the intensity of ^5^D_0_ → ^7^F_4_ transition at 77 K temperature of 75% Eu^3+^ doped sample is much stronger than the one in 1% Eu^3+^ doped sample at the same temperature. In order to calculate the TQ_1/2_ (temperature at which phosphor loses half of its efficiency) values, the Boltzmann sigmoidal^39^ fit was performed to the normalized emission intensity data:


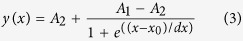


Here *y*(*x*) is the normalized emission intensity value at a given *x* (temperature in K in this case), *A*_*1*_ and *A*_*2*_ are the initial value (left horizontal asymptote) and the final value (right horizontal asymptote), respectively. *x*_*0*_ (*x*_*0*_ = TQ_1/2_) is the centre of the sigmoid, and *dx* is the change in *x* corresponding to the most significant change in *y*(*x*) values. Since the fitting was performed on the normalized (divided by maximum intensity at 77 K for 613 nm line) emission intensity, the *A*_*1*_ and *A*_*2*_ values were set to 1 and 0, respectively. The obtained fitting lines and calculated TQ_1/2_ values for the samples doped with 1%, 10%, and 75% Eu^3+^ are given in Fig. 5c. The intensity of 613 nm line decreases gradually with increasing the temperature regardless the Eu^3+^ concentration. The calculated TQ_1/2_ values for 1%, 10%, and 75% Eu^3+^ doped samples are 295, 349, and 449 K, respectively. This shows that samples with low europium concentration suffers more from the thermal quenching. However, even though the emission intensity at elevated temperatures decreases, the emission lines of Eu^3+^ tend to broaden. Therefore, the total decrease of light output might be smaller than predicted from emission intensity change. Thus, the better way to calculate TQ_1/2_ values is by using emission integrals. The Boltzmann fit data of normalized emission integrals of 1%, 10%, and 75% Eu^3+^ doped samples is shown in Fig. 5d and the derived TQ_1/2_ values are 397, 457, and 515 K, respectively. It is obvious that the obtained TQ_1/2_ values are much higher than those calculated from emission intensity. Moreover, the emission integral method is also insensitive to the peak position change upon increasing temperature.

From the temperature dependent emission spectra the activation energy according to the single barrier quenching model can also be derived^40^,^41^,^42^:


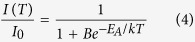


Here *I*(*T*) and *I*_*0*_ are temperature dependent emission integral and highest value of emission integral, respectively. *E*_*A*_ is activation energy, *k* is Boltzmann constant (8.617342·10^−5 ^eV/K)^43^, and *T* is temperature in K. *B* is the quenching frequency factor, which can also be expressed as *Γ*_*0*_/*Γ*_*υ*_, where *Γ*_*0*_ is the attempt rate of the non-radiative process and *Γ*_*υ*_ is the radiative rate. The activation energies for 1%, 10%, and 75% Eu^3+^ doped samples are 0.15 ± 0.01 eV, 0.29 ± 0.02 eV, and 0.42 ± 0.12 eV, respectively. The obtained *E*_*A*_ values are in good agreement with the respective TQ_1/2_ values, i.e. they both increase with increasing Eu^3+^ concentration in the Y_2_Mo_4_O_15_ lattice. The equation 4 can also be utilized to calculate the TQ_1/2_ values of the phosphor. One can easily derive that the TQ_1/2_ expression based on equation 4 is^42^:


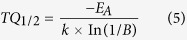


The *E*_*A*_ values are given above and the *B* values for the samples doped with 1%, 10%, and 75% Eu^3+^ are 89, 1506, and 11742. Thus the calculated respective TQ_1/2_ values are 388, 460, and 520 K. These temperatures are very close to the ones obtained from the Boltzmann fit, therefore, both methods can be used for the TQ_1/2_ determination.

**Figure 6:**
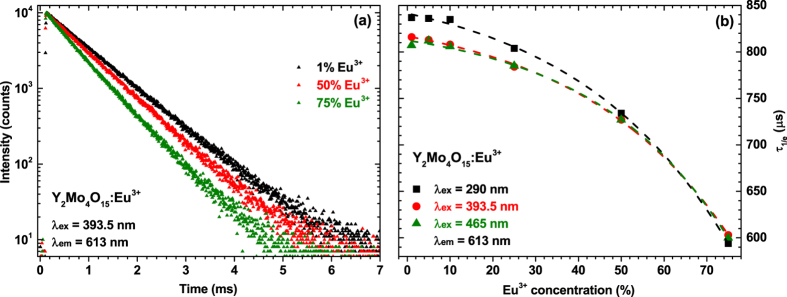
Photoluminescence decay curves and calculated decay constants. (**a**) Photoluminescence decay (λ_ex_ = 393.5 nm, λ_em_ = 613 nm) curves of Y_2_Mo_4_O_15_:Eu^3+^ samples doped with 1%, 50% and 75% Eu^3+^. (**b**) Decay constants of Y_2_Mo_4_O_15_:Eu^3+^ phosphors as a function of Eu^3+^ concentration and excitation wavelength.

Figure 6a shows decay curves of 1%, 50%, and 75% Eu^3+^ doped samples when specimens were excited at 393.5 nm and emission monitored at 613 nm. Decay curves for all Eu^3+^ concentrations are linear implying only one depopulation mechanism of ^5^D_0_ level. The increasing europium concentration in the Y_2_Mo_4_O_15_ host lattice resulted in steeper decay curves indicating the decrease of luminescence lifetimes. All decay curves were fitted employing a single exponential decay function:


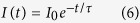


where *I*(*t*) is the intensity at a given time *t*, *I*_*0*_ is initial intensity and *τ* is a decay constant (lifetime)^35^. The obtained decay constants as a function of Eu^3+^ concentration and excitation wavelength are depicted in Fig. 6b. Excitation of samples at 393.5 nm (^7^F_0_ → ^5^L_6_ transition) and 465 nm (^7^F_0_ → ^5^D_2_ transition) yields the same decay constants regardless the Eu^3+^ concentration, what shows that radiationless decay from the higher energy levels (^5^L_6_ and ^5^D_2_) to the ^5^D_0_ level, from which the luminescence occurs, is very fast and of the same order. Excitation of samples through CT band gives slightly larger values of decay constant at Eu^3+^ concentrations up to 25%. However, at higher activator concentrations decay constants levels. The exact calculated photoluminescence decay values with standard deviations as a function of Eu^3+^ concentration and excitation wavelength are tabulated in Table s2.

**Figure 7:**
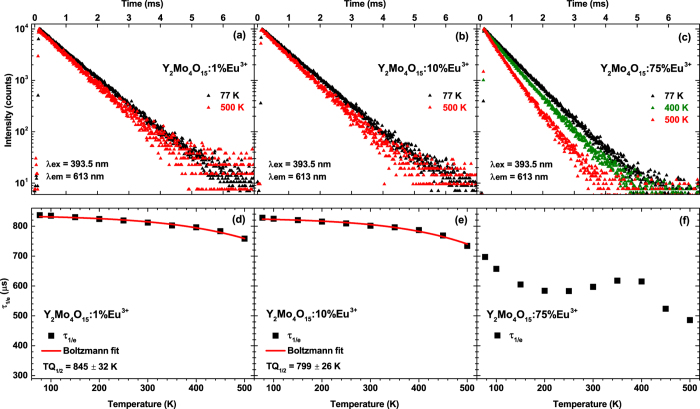
Temperature dependent photoluminescence decay curves and calculated decay constants. Decay (λ_ex_ = 393.5 nm, λ_em_ = 613 nm) curves of (**a**) Y_2_Mo_4_O_15_:1%Eu^3+^, (**b**) Y_2_Mo_4_O_15_:10%Eu^3+^, (**c**) Y_2_Mo_4_O_15_:75%Eu^3+^. Calculation of TQ_1/2_ values for the samples doped with (**d**) 1% Eu^3+^ and (**e**) 10% Eu^3+^. (**f**) Temperature dependent decay constants of 75% Eu^3+^ doped sample.

A better insight to the thermal quenching processes might be gained by measuring temperature dependent decay curves. Such curves for samples doped with 1%, 10%, and 75% Eu^3+^ are shown in Fig. 7a–c, respectively. The decay curves for all samples become steeper with increasing temperature, thus the photoluminescence lifetime gets shorter. However, the change of decay constants of samples doped with 1% and 10% Eu^3+^ is rather small, whereas for the 75% Eu^3+^ doped sample this change is quite substantial. The calculated photoluminescence lifetimes as a function of temperature were also fitted with Boltzmann function in order to calculate the TQ_1/2_. The obtained results are shown in Fig. 7d–f. Unfortunately, it was not possible to fit the data obtained for the sample doped with 75% Eu^3+^, since the lifetimes as a function of temperature distributes in rather bizarre way and the Boltzmann fit did not converge. The calculated TQ_1/2_ values for 1% and 10% Eu^3+^ doped samples are 845 ± 32 K and 799 ± 26 K, respectively. The relatively high standard deviation values are obtained due to the fact, that the fitting function do not reach the turning point. It is also clear that the calculated TQ_1/2_ values from the temperature dependent lifetime data are much larger if compared to ones obtained from temperature dependent emission integrals. Moreover, the smaller decrease of lifetime values upon temperature increase is evident. This implies that the internal quantum yield (IQY) decreases slower than the external quantum yield (EQY). The internal quantum yield of the phosphor can be expressed as^25^:


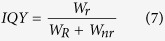


where *W*_*r*_ and *W*_*nr*_ are the probabilities of radiative and non-radiative transitions, respectively. The sum of radiative and non-radiative probabilities is unity, therefore, the decreasing IQY is related to the increased probability of *W*_*nr*_. The internal quantum yield is dependent only on the activator ion and its local surrounding. The external quantum yield can be expressed as^25^:


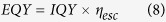


where *η*_*esc*_ is the escape efficiency of the photons from the phosphor particle. The faster decrease of EQY in our case is related to the decreasing photon escape efficiency. All calculated photoluminescence decay values with standard deviations as a function of temperature are listed in Table s3.

**Figure 8:**
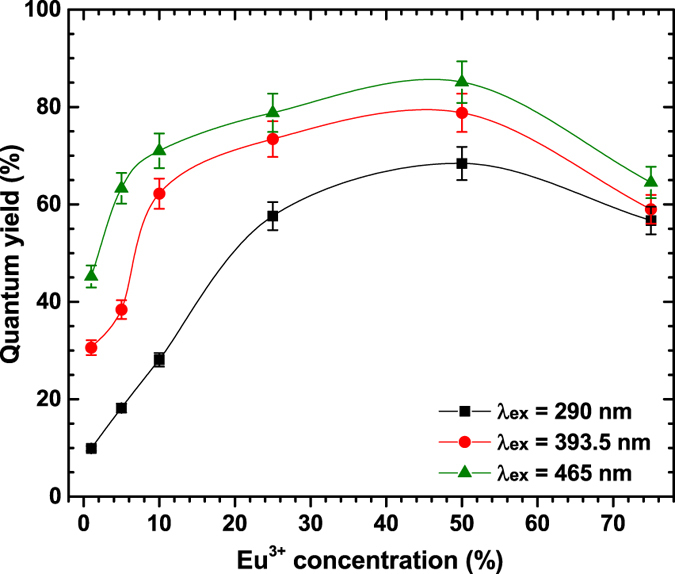
Quantum yields of Y_2_Mo_4_O_15_:Eu^3+^ phosphors as a function of Eu^3+^ concentration and excitation wavelength. The lines are drawn to guide the eye.

The calculated external quantum yields for Y_2_Mo_4_O_15_:Eu^3+^ phosphor samples under the three excitation wavelengths are depicted in Fig. 8. The quantum yields reaches top values when 50% of Eu^3+^ is introduced into the host lattice. Further increase of Eu^3+^ concentration leads to the decrease of quantum yield. This decrease is probably caused by concentration quenching. Since the quenching concentration is rather high it can be concluded that [MoO_4_]^2−^ and [Mo_2_O_7_]^2−^ groups that are present in the structure effectively shields Eu^3+^ ions from each other, thus, preventing the energy transfer between Eu^3+^ pairs. The same pattern was observed for all three excitation wavelengths. Moreover, the efficiency increases with increasing excitation wavelength (decreasing energy). The maximum efficiency (ca. 85%) was obtained for the sample doped with 50% of Eu^3+^ and excited at 465 nm. The mentioned energy transfer between Eu^3+^ pairs is strongly dependent on the average distance (*R*_*c*_) between the Eu^3+^ ions. Therefore, knowing the critical concentration of Eu^3+^ it is possible to calculate this distance^44^:


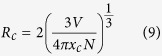


where *V* is the volume of the crystallographic unit cell, *x*_*c*_ is the critical concentration and *N* is the number of lattice sites that can be occupied by activator ions. In our case *V* = 672.74 Å^3^ (see Table s1), *x*_*c*_ is 75%, i.e. 0.75 and *N* = 4, thus the obtained *R*_*c*_ value is equal to 7.54 Å.

In order to demonstrate the emission colour dependency of the synthesized phosphors, the CIE chromaticity coordinates in 1931 and 1976 colour space diagrams were calculated. Table s4 shows the calculated colour coordinates as a function of Eu^3+^ concentration and excitation wavelength, whereas colour coordinates as a function of temperature of samples doped with 1%, 10%, and 75% Eu^3+^ are given in Table s5. Besides the colour coordinates, the luminous efficacy (LE) (a parameter describing how bright the radiation is perceived by the average human eye) values were also calculated from the respective emission spectra by using the following equation^4^:


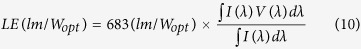


**Figure 9:**
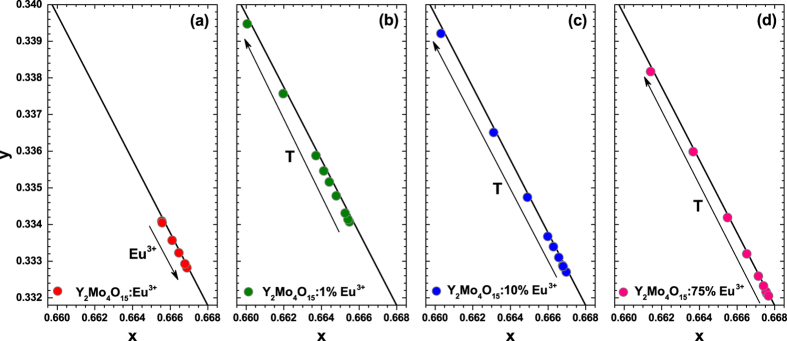
Fragments of the CIE 1931 colour diagram. The colour points of (**a**) Y_2_Mo_4_O_15_:Eu^3+^ as a function of Eu^3+^ concentration and as a function of temperature of (**b**) 1%, (**c**) 10%, and (**d**) 75% Eu^3+^ doped samples. All samples were excited at 393.5 nm.

Here *I*(*λ*) is the emission spectrum of the phosphor and *V*(*λ*) is the human eye sensitivity curve. The human eye sensitivity curve possesses a maxima at 555 nm, therefore, the highest possible LE value (683 lm/W_opt_) is obtained for monochromatic green radiation at 555 nm. The obtained LE values were around 242 lm/W_opt_ for all Eu^3+^ concentrations regardless the excitation wavelength. These values are rather high and can be compared to such well known red emitting phosphors as Sr_2_Si_5_N_8_:Eu^2+^ (λ_em_ = 620 nm, LE = 240 lm/W_opt_), CaAlSiN_3_:Eu^2+^ (λ_em_ = 650 nm, LE = 150 lm/W_opt_), CaS:Eu^2+^ (λ_em_ = 650 nm, LE = 85 lm/W_opt_)^45^. The LE values increased from 231 to around 242 lm/W_opt_ when the temperature was increased from 77 to 500 K. This shift goes hand in hand with the shift of colour coordinates (see Fig. 9b–d) to the direction of orange region when temperature is increased. The so-called blue shift of emission results in higher overlap between emission spectra and human eye sensitivity curve thus leading to larger LE values.

Fragments of the CIE 1931 colour space diagrams with the colour coordinates of Y_2_Mo_4_O_15_:Eu^3+^ phosphors as a function of Eu^3+^ concentration are shown in Fig. 9a. The colour coordinates are relatively stable with respect to Eu^3+^ concentration, however, the tendency is that they slightly shift towards deeper red region if the Eu^3+^ concentration increases. Colour coordinates are also very close to the edge of the colour space diagram indicating the high colour purity of the prepared phosphors.

**Figure 10:**
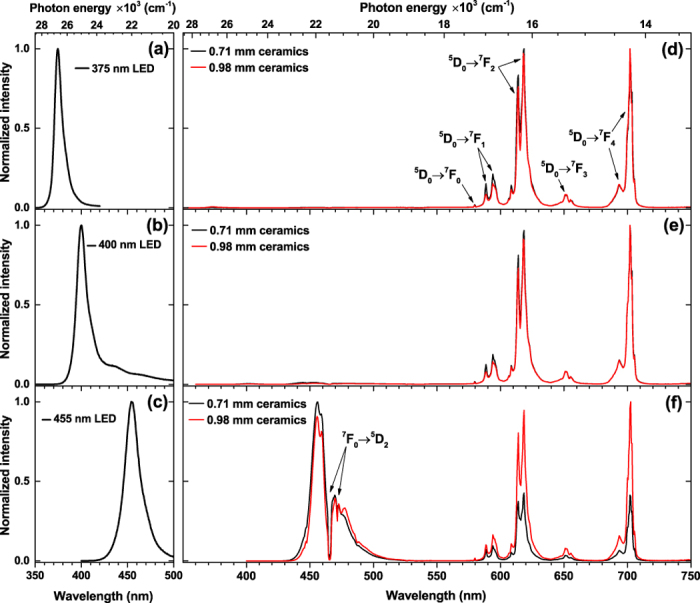
Emission spectra of LEDs and ceramics. (**a**) 375 nm LED, (**b**) 400 nm LED, and (**c**) 455 nm LED. Emission spectra of Y_2_Mo_4_O_15_:75%Eu^3+^ ceramic disks excited with (**d**) 375 nm LED, (**e**) 400 nm LED, and (**f**) 455 nm LED.

**Figure 11:**
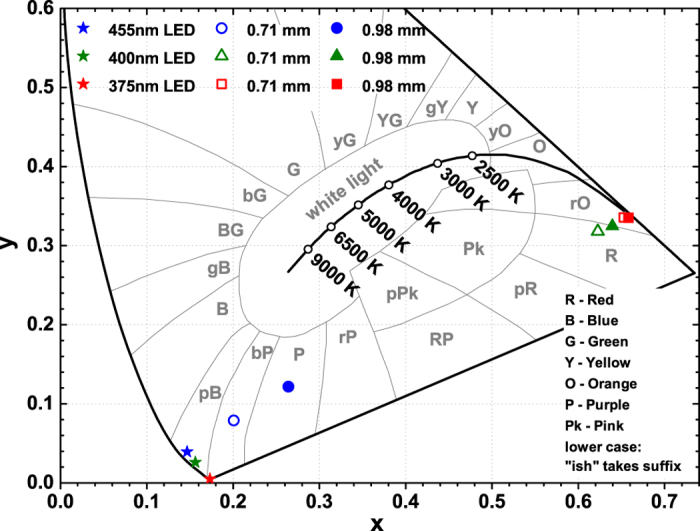
Fragment of the CIE 1931 colour diagram. The colour points of Y_2_Mo_4_O_15_:75Eu^3+^ ceramics combined with 375, 400, and 455 nm LEDs. Stars, open, and filled symbols denote colour points of LEDs, LEDs with 0.71 mm ceramic disk, and LEDs with 0.98 mm ceramic disk, respectively.

Due to the spin and parity forbidden character of their [Xe]4f^n^ → [Xe]4f^n^ transitions, lanthanide ions often suffers from weak absorption^5^,^45^,^46^. One way to increase the absorption strength of the phosphor is preparing the ceramics. In such way the pathway of the incident photons increases, thus increasing the probability of absorption of these photons. The ceramic disks of Y_2_Mo_4_O_15_:75%Eu^3+^ sample with the thickness of 0.71 and 0.98 mm (ρ = 2.93 g/cm^3^) were prepared and their optical properties investigated. These disks were placed on the top of three LEDs with the emission maximum at 375, 400, and 455 nm, *i.e*. the emission spectra were recorded in the transmission mode. The respective emission spectra of mentioned LEDs are shown in Fig. 10a–c. the emission spectra of ceramic disks excited by LEDs are shown in Fig. 10d–f. It is obvious that ceramic disks of both thicknesses absorb all radiation emitted by 375 and 400 nm LEDs and convert it into the red light. Therefore, even thinner ceramics could be used. On the other hand, preparation of thinner ceramic disks with available equipment was rather complicated. This strong absorption in the mentioned spectral regions is possible due to the abundance of Eu^3+^ absorption lines in these spectral regions. The situation, however, is different if disks are excited with 455 nm LED. In this spectral region there is only one ^7^F_0_ → ^5^D_2_ transition with narrow absorption lines at around 465 nm. Thus, a large portion of the blue light emitted by LED passes through ceramic disk without being absorbed. This is visible in emission spectra depicted in Fig. 10f, where absorption lines of Eu^3+^ due ^7^F_0_ → ^5^D_2_ transition are visible in the region of emission spectrum of 455 nm LED. Obviously, the 455 nm LED is not suitable for gaining red light, however, various tints of pink light might be obtained as shown in Fig. 11. It is also interesting to note that the emission spectra of ceramic disks are a little bit different if compared to the powder samples, namely, the five narrow peaks of ^5^D_0_ → ^7^F_2_ transition merge into the three broader peaks and the emission line originating from ^5^D_0_ → ^7^F_4_ transition become even stronger. The latter also slightly increases with the increase of ceramic disk thickness. Considering this ceramics for colour conversion, however, such increase in emission intensity at around 700 nm is undesirable, since the human eye sensitivity at this wavelength is very low and this would reduce the luminous efficacy of the prepared light source.

The calculated colour coordinates in CIE 1931 colour space of light sources obtained by combining 375, 400, and 455 nm LEDs and Y_2_Mo_4_O_15_:75%Eu^3+^ ceramic disks of both thicknesses are depicted in Fig. 11. The colour points of LEDs are also included for the reference. The exact values are tabulated in Table s6 together with calculated luminous efficacies. The colour coordinates of light source obtained from 375 nm LED and Y_2_Mo_4_O_15_:75%Eu^3+^ ceramics are close to the edge of the colour space diagram, therefore, the colour purity is high. However, the colour purity decreases if 400 nm LED is used as an excitation source. It is possible that small amount of violet light emitted by LED passes through the ceramic disks thus reducing the colour purity. As was already mentioned the combination of 455 nm LED and prepared ceramic disks have not yielded the red colour. On the other hand, one combining this LED with ceramic disks could obtain the light source with the colours ranging from bluish purple to purple or even reddish purple depending on the thickness of ceramics. The calculated luminous efficacy values were in the range of 200–184 lm/W_opt_ for light sources prepared employing 375 and 400 nm LEDs. The slightly lower values if compared to the powder samples are obtained due to the increased emission intensity in deep red spectral region at around 700 nm. Using 455 nm LED as an excitation source yielded substantially lover LE values in the range of 94–121 lm/W_opt_. This value increase with increasing thickness of the ceramics due to decreasing intensity of blue light passing through the disk unabsorbed.

## Conclusions

Single phase Y_2_Mo_4_O_15_:Eu^3+^ phosphors with Eu^3+^ concentration in the range of 1–75% were prepared by high temperature solid state reaction. Samples showed bright red luminescence under excitation of near-UV and blue radiation. These phosphors possess high colour purity, excellent thermal stability and high quantum yields. Temperature dependent photoluminescence decay measurements indicated that internal quantum yield decreases much slower than the external one what was related to the decreasing photon escape efficiency from the particle upon increasing the temperature. The combination of Y_2_Mo_4_O_15_:75%Eu^3+^ ceramic disks with 375, and 400 nm LEDs yielded red light sources and with 455 nm LED resulted in various tints of purple colour.

## Additional Information

**How to cite this article**: Janulevicius, M. *et al*. Luminescence and luminescence quenching of highly efficient Y_2_Mo_4_O_15_:Eu^3+^ phosphors and ceramics. *Sci. Rep*. **6**, 26098; doi: 10.1038/srep26098 (2016).

## Supplementary Material

Supplementary Information
