# Supplementary material for: Luminescence and luminescence quenching of highly efficient Y2Mo4O15:Eu3+ phosphors and ceramics
Source: Sci Rep. 2016 May 16;6:26098. doi: 10.1038/srep26098 (PMC4867634; doi:10.1038/srep26098)
Supplement: Supplementary Information [file srep26098-s1.pdf]

Supplementary Information

Luminescence and luminescence quenching of highly efficient $Y_2Mo_4O_{15}:Eu^{3+}$ phosphors and ceramics

Matas Janulevicius[a], Paulius Marmokas[a], Martynas Misevicius[b], Julija Grigorjevaite[a], Lina Mikoliunaite[c], Simas Sakirzanovas[d,e], Arturas Katelnikovas[a,*]

[a] *Department of Analytical and Environmental Chemistry, Vilnius University, Naugarduko 24, LT-03225 Vilnius, Lithuania*

[b] *Department of Inorganic Chemistry, Vilnius University, Naugarduko 24, LT-03225 Vilnius, Lithuania*

[c] *Department of Physical Chemistry, Vilnius University, Naugarduko 24, LT-03225 Vilnius, Lithuania*

[d] *Department of Applied Chemistry, Vilnius University, Naugarduko 24, LT-03225 Vilnius, Lithuania*

[e] *Institute of Chemistry, Centre for Physical Sciences and Technology, A. Gostauto 9, LT-01108 Vilnius, Lithuania*

[*] *Corresponding author: Tel: +370 697 23123; E-mail: arturas.katelnikovas@chf.vu.lt*

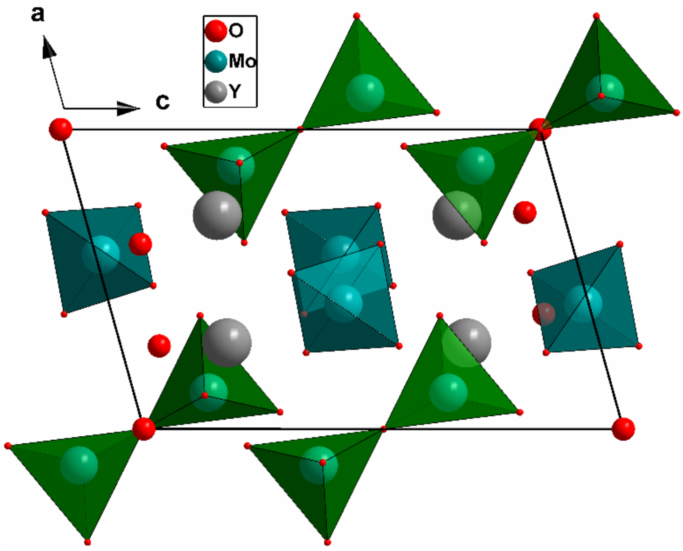

Figure s1. Unit cell of $Y_2Mo_4O_{15}$ along the b-axis.

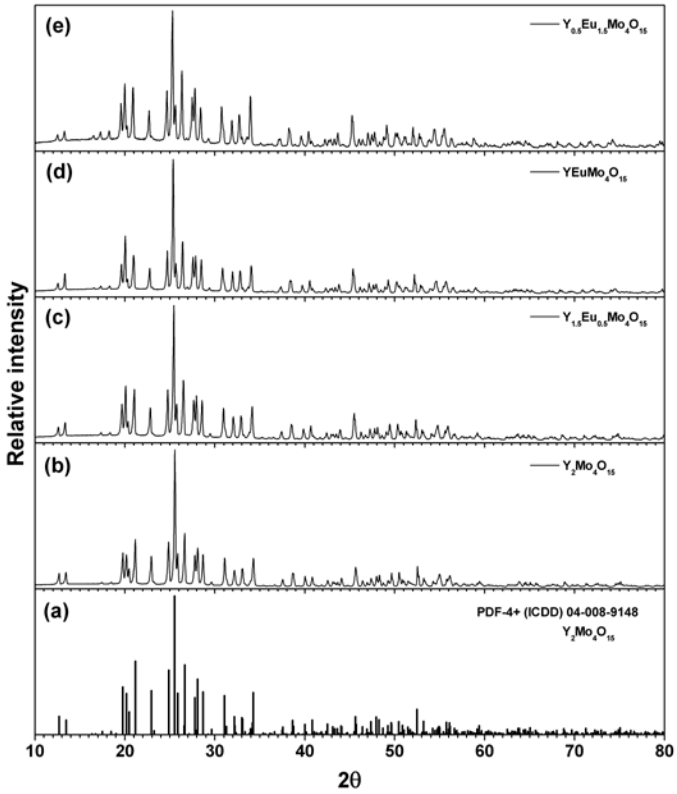

Figure s2. (a) XRD reference pattern of $Y_2Mo_4O_{15}$. Powder XRD diffraction patterns of $Y_2Mo_4O_{15}:Eu^{3+}$ samples doped with (b) 0% Eu^{3+}, (c) 25% Eu^{3+}, (d) 50% Eu^{3+}, (e) 75% Eu^{3+}.

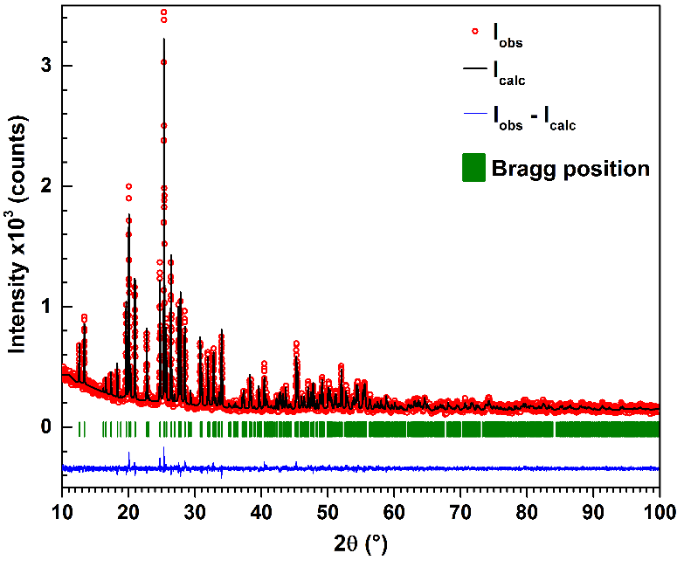

Figure s3. Rietveld refinement of $Y_2Mo_4O_{15}$:75%Eu^{3+} powder XRD pattern.

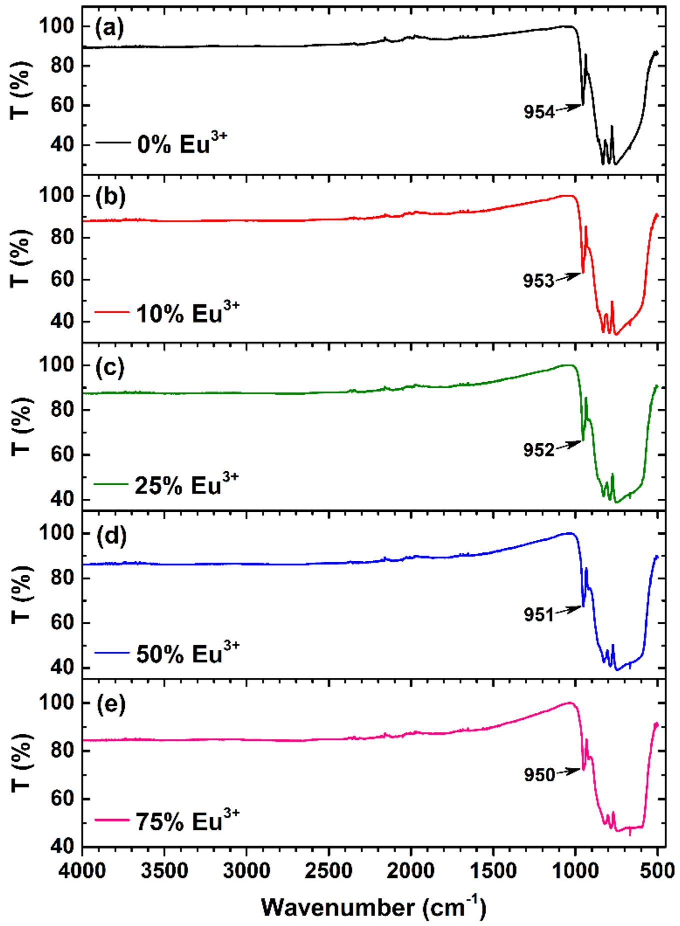

Figure s4. FTIR spectra of $Y_2Mo_4O_{15}:Eu^{3+}$ doped with (a) 0% Eu^{3+}, (b) 10% Eu^{3+}, (c) 25% Eu^{3+}, (d) 50% Eu^{3+} and (e) 75% Eu^{3+}.

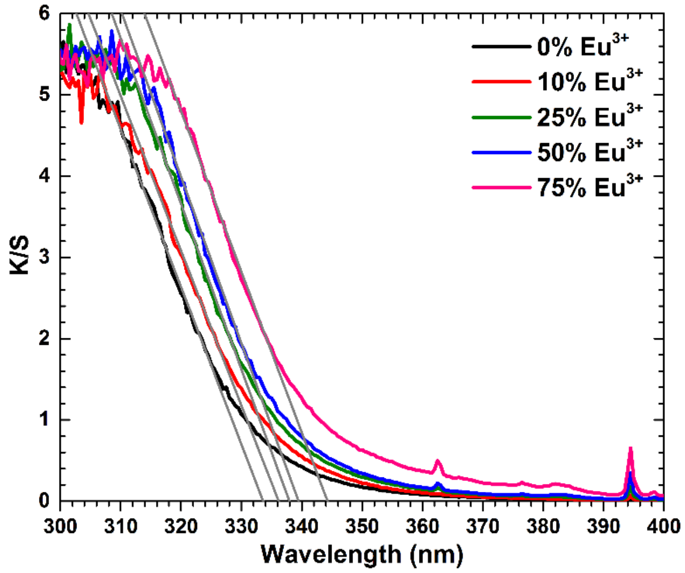

Figure s5. Absorption spectra of $Y_2Mo_4O_{15}:Eu^{3+}$ as a function of Eu^{3+} concentration.

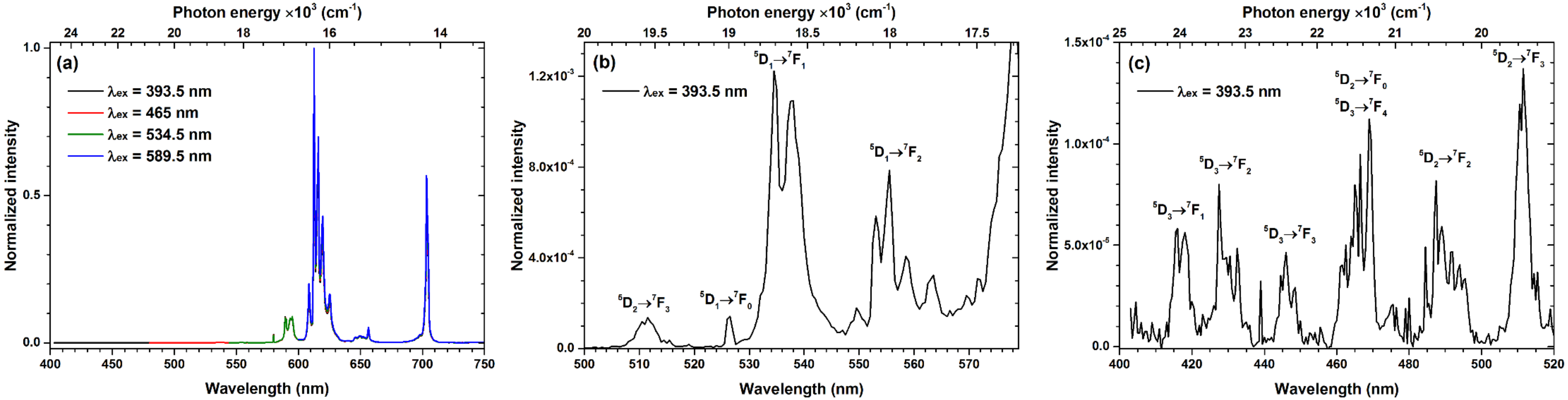

Figure s6. (a) Emission spectra of $Y_2Mo_4O_{15}$:50%Eu^{3+} sample excited at different wavelengths; (b) and (c) magnified high energy regions of emission spectra (λ_{ex} = 393.5 nm).

Table s1. Unit cell parameters of $Y_2Mo_4O_{15}:Eu^{3+}$ derived from the Rietveld refinement of the powder XRD patterns.

Eu^{3+} (%)	a (Å)	b (Å)	c (Å)	V (Å^3)	β (°)
**0**	6.8189	9.5787	10.5185	661.92	105.54
**5**	6.8213	9.5846	10.5232	662.82	105.55
**10**	6.8256	9.5908	10.5292	663.99	105.57
**25**	6.8371	9.6088	10.5452	667.22	105.61
**50**	6.8561	9.6404	10.5719	672.74	105.69
**75**	6.8786	9.6751	10.6033	679.15	105.75

Table s2. Photoluminescence decay values of $Y_2Mo_4O_{15}:Eu^{3+}$ phosphors as a function of Eu^{3+} concentration and excitation wavelength (λ_{em} = 613 nm).

Eu^{3+} (%)	λ_{ex} = 290 nm		λ_{ex} = 393.5 nm		λ_{ex} = 465 nm	
	τ (μs)	Std. dev. (μs)	τ (μs)	Std. dev. (μs)	τ (μs)	Std. dev. (μs)
**1**	837	0.5	816	0.4	807	0.4
**5**	836	0.5	813	0.4	812	0.4
**10**	836	0.5	808	0.4	806	0.4
**25**	804	0.5	784	0.4	785	0.4
**50**	734	0.4	727	0.4	727	0.4
**75**	592	0.4	601	0.4	600	0.4

Table s3. Temperature dependent photoluminescence decay (λ_{ex} = 393.5 nm, λ_{em} = 613 nm) values of $Y_2Mo_4O_{15}:Eu^{3+}$ phosphors doped with 1%, 10% and 75% Eu^{3+}.

T (K)	$Y_2Mo_4O_{15}$:1%Eu^{3+}		$Y_2Mo_4O_{15}$:10%Eu^{3+}		$Y_2Mo_4O_{15}$:75%Eu^{3+}	
	τ (μs)	Std. dev. (μs)	τ (μs)	Std. dev. (μs)	τ (μs)	Std. dev. (μs)
**77**	837	0.6	828	0.6	697	0.5
**100**	835	0.6	825	0.6	657	0.5
**150**	830	0.5	820	0.6	605	0.4
**200**	824	0.5	815	0.5	584	0.4
**250**	819	0.5	809	0.5	583	0.4
**300**	812	0.6	801	0.5	597	0.4
**350**	802	0.7	796	0.6	618	0.4
**400**	796	0.8	787	0.6	615	0.4
**450**	783	0.9	769	0.7	524	0.4
**500**	758	1.0	734	0.9	486	0.5

Table s4. Colour coordinates (CIE 1931 and CIE 1976 colour space) and luminous efficacies (LE) of synthesized phosphors as a function of Eu^{3+} concentration and excitation wavelength.

Eu^{3+} (%)	λ_{ex} = 290 nm					λ_{ex} = 393.5 nm					λ_{ex} = 465 nm				
	CIE 1931		CIE 1976		LE (lm/W_{opt})	CIE 1931		CIE 1976		LE (lm/W_{opt})	CIE 1931		CIE 1976		LE (lm/W_{opt})
	x	y	u’	v’		x	y	u’	v’		x	y	u’	v’	
**1**	0.6615	0.3379	0.4617	0.5306	237	0.6656	0.3341	0.4689	0.5296	241	0.6650	0.3347	0.4678	0.5297	241
**5**	0.6654	0.3342	0.4686	0.5296	243	0.6656	0.3341	0.4689	0.5295	243	0.6636	0.3356	0.4656	0.5299	243
**10**	0.6663	0.3334	0.4702	0.5294	242	0.6661	0.3336	0.4699	0.5294	242	0.6654	0.3341	0.4687	0.5296	242
**25**	0.6670	0.3327	0.4715	0.5292	240	0.6665	0.3332	0.4705	0.5293	241	0.6664	0.3332	0.4705	0.5293	242
**50**	0.6672	0.3325	0.4719	0.5291	239	0.6669	0.3328	0.4713	0.5292	241	0.6671	0.3327	0.4716	0.5292	241
**75**	0.6672	0.3326	0.4718	0.5292	239	0.6668	0.3329	0.4711	0.5293	241	0.6669	0.3328	0.4713	0.5292	241

Table s5. Colour coordinates (CIE 1931 and CIE 1976 colour space) and luminous efficacies (LE) of synthesized phosphors as a function of Eu^{3+} concentration and temperature (λ_{ex} = 393.5 nm).

T (K)	$Y_2Mo_4O_{15}$:1%Eu^{3+}					$Y_2Mo_4O_{15}$:10%Eu^{3+}					$Y_2Mo_4O_{15}$:75%Eu^{3+}				
	CIE 1931		CIE 1976		LE (lm/W_{opt})	CIE 1931		CIE 1976		LE (lm/W_{opt})	CIE 1931		CIE 1976		LE (lm/W_{opt})
	x	y	u’	v’		x	y	u’	v’		x	y	u’	v’	
**77**	0.6642	0.3351	0.4667	0.5298	231	0.6665	0.3331	0.4707	0.5293	231	0.6677	0.3320	0.4729	0.5290	229
**100**	0.6651	0.3343	0.4682	0.5296	231	0.6674	0.3322	0.4724	0.5290	231	0.6684	0.3313	0.4741	0.5288	229
**150**	0.6646	0.3348	0.4674	0.5297	231	0.6669	0.3328	0.4713	0.5292	231	0.6678	0.3320	0.4729	0.5290	229
**200**	0.6642	0.3353	0.4666	0.5299	232	0.6667	0.3330	0.4710	0.5292	231	0.6676	0.3321	0.4727	0.5290	231
**250**	0.6641	0.3354	0.4664	0.5299	234	0.6667	0.3330	0.4709	0.5293	231	0.6675	0.3323	0.4724	0.5291	231
**300**	0.6639	0.3356	0.4659	0.5300	234	0.6666	0.3331	0.4708	0.5293	232	0.6672	0.3325	0.4719	0.5291	232
**350**	0.6632	0.3363	0.4647	0.5302	237	0.6663	0.3334	0.4701	0.5294	233	0.6667	0.3331	0.4709	0.5293	233
**400**	0.6613	0.3382	0.4612	0.5307	237	0.6651	0.3346	0.4680	0.5297	235	0.6654	0.3343	0.4685	0.5296	234
**450**	0.6578	0.3415	0.4550	0.5315	243	0.6628	0.3368	0.4639	0.5303	238	0.6636	0.3361	0.4652	0.5301	237
**500**	0.6527	0.3464	0.4462	0.5328	247	0.6603	0.3392	0.4594	0.5309	240	0.6611	0.3385	0.4607	0.5308	241

Table s6. Colour coordinates (CIE 1931 and CIE 1976 colour space) and luminous efficacies (LE) of different thickness $Y_2Mo_4O_{15}$:75%Eu^{3+} ceramics mounted on 375, 400, and 455 nm LEDs.

**Thickness (mm)**	**LED (nm)**	**CIE 1931**		**CIE 1976**		**LE (lm/W_{opt})**
		**x**	**y**	**u'**	**v'**	
0.71	375	0.6526	0.3357	0.4561	0.5279	200
	400	0.6226	0.3181	0.4470	0.5138	187
	455	0.2007	0.0791	0.2264	0.2006	94
0.98	375	0.6582	0.3352	0.4614	0.5287	189
	400	0.6396	0.3248	0.4554	0.5203	184
	455	0.2641	0.1217	0.2686	0.2785	121
